# Luteolin, a Potent Anticancer Compound: From Chemistry to Cellular Interactions and Synergetic Perspectives

**DOI:** 10.3390/cancers14215373

**Published:** 2022-10-31

**Authors:** Hardeep Singh Tuli, Prangya Rath, Abhishek Chauhan, Katrin Sak, Diwakar Aggarwal, Renuka Choudhary, Ujjawal Sharma, Kanupriya Vashishth, Sheetu Sharma, Manoj Kumar, Vikas Yadav, Tejveer Singh, Mukerrem Betul Yerer, Shafiul Haque

**Affiliations:** 1 Department of Biotechnology, Maharishi Markandeshwar Engineering College, Maharishi Markandeshwar (Deemed to be University), Mullana-Ambala 133207, India; 2 Amity Institute of Environmental Sciences, Amity University, Noida 201303, India; 3 Amity Institute of Environmental Toxicology, Safety and Management, Amity University, Noida 201303, India; 4 NGO Praeventio, 50407 Tartu, Estonia; 5 Department of Human Genetics and Molecular Medicine, Central University of Punjab, Bhatinda 151001, India; 6 Department of Cardiology, Advance Cardiac Centre, Post Graduate Institute of Medical Education and Research (PGIMER), Chandigarh 160012, India; 7 Department of Pharmacovigilace and Clinical Research, Chitkara University, Rajpura 140401, India; 8 Department of Chemistry, Maharishi Markandeshwar University Sadopur, Ambala 133001, India; 9 Department of Translational Medicine, Clinical Research Centre, Skåne University Hospital, Lund University, SE-20213 Malmö, Sweden; 10 Translational Oncology Laboratory, Department of Zoology, Hansraj College, Delhi University, Delhi 110007, India; 11 Department of Pharmacology, Faculty of Pharmacy, Erciyes University, Kayseri 38039, Turkey; 12 Research and Scientific Studies Unit, College of Nursing and Allied Health Sciences, Jazan University, Jazan 45142, Saudi Arabia

**Keywords:** luteolin, apoptosis and cell cycle, anti-metastasis, anti-inflammation, synergistic action

## Abstract

**Simple Summary:**

Common flavonoid luteolin 3′,4′,5,7-tetrahydroxyflavone has immense potential to be utilized as a chemopreventive dietary molecule. According to available data, luteolin interacts with a number of known cellular targets and prevents the growth of cancer cells by triggering apoptosis and cell cycle arrest. Inhibiting tumor cell metastasis and angiogenesis is another promising function of luteolin, according to recent research. Luteolin has also been discovered to be a good option for synergistic investigations and may be able to reverse cancer cells’ medication resistance. The current review focuses on the work being done to find molecular targets of luteolin in cancer. Additionally, the use of luteolin combinations and delivery systems enabled by nanotechnology are presented. The review is distinctive by offering all potential cellular targets of luteolin in cancer on one platform. The text is accompanied by excellent visual aids.

**Abstract:**

Increasing rates of cancer incidence and the toxicity concerns of existing chemotherapeutic agents have intensified the research to explore more alternative routes to combat tumor. Luteolin, a flavone found in numerous fruits, vegetables, and herbs, has exhibited a number of biological activities, such as anticancer and anti-inflammatory. Luteolin inhibits tumor growth by targeting cellular processes such as apoptosis, cell-cycle progression, angiogenesis and migration. Mechanistically, luteolin causes cell death by downregulating Akt, PLK-1, cyclin-B1, cyclin-A, CDC-2, CDK-2, Bcl-2, and Bcl-xL, while upregulating BAX, caspase-3, and p21. It has also been reported to inhibit STAT3 signaling by the suppression of STAT3 activation and enhanced STAT3 protein degradation in various cancer cells. Therefore, extensive studies on the anticancer properties of luteolin reveal its promising role in chemoprevention. The present review describes all the possible cellular interactions of luteolin in cancer, along with its synergistic mode of action and nanodelivery insight.

## 1. Introduction

The identification of novel and efficient therapies for management of cancer is very important, considering the continuously increasing incidence of malignant diseases all over the world. It is expected that during the next two decades the global cancer burden will reveal about a 50% rise, reaching 28.4 million new cases by 2040 [1]. Prediction of such a high incidence rate definitely requires more intense implementation of efficient countermeasures, including the development of novel potent anticancer drugs. The plant kingdom has already been an abundant resource for natural remedies used in the form of herbal extracts to treat both benign as well as malignant neoplasms for centuries [2]. Only more recently, the structural features and mechanistic insights of such bioactive phytochemicals have been made the subject of in-depth preclinical investigations. In fact, in 1960, the United States National Cancer Institute (NCI) launched the screening program for anticancer properties of plant-derived products, leading to identification of several new compounds, such as vincristine, vinblastine and paclitaxel that are currently used as chemotherapeutic drugs in the clinical settings [3]. These studies laid a strong foundation for subsequent intensive exploration of natural anticancer agents, clearly demonstrating that the lead structures provided by nature and synthesized by plants might be highly valuable for developing novel efficient drugs against cancer. Hence, investigation into such bioactive phytochemicals represents a very attractive research field.

Among natural plant-derived compounds, one of the most explored plant-derived chemicals is the flavone luteolin (3′,4′,5,7-tetrahydroxyflavone) that occurs widely in diverse vegetables and herbs such as cabbage, kale, lettuce, thyme, parsley, rosemary and oregano [4]. A number of recent studies have demonstrated the ability of luteolin to suppress the carcinogenesis process by perturbing the cell cycle progression, inhibiting proliferation, promoting apoptosis, and restricting migration and invasion of cancerous cells [5,6]. For example, luteolin can retard malignant progression in breast cancer [7,8], colorectal cancer [9,10], lung cancer [11] and prostate cancer models [12], among others. Such anticancer activities of luteolin are regulated through its ability to interact with different molecular target sites and modulate a variety of signaling cascades in tumor cells [13,14].

In the current comprehensive review article, the chemical composition and bioavailability of luteolin in human beings are discussed, besides describing the different anticancer activities of this flavone. Precisely, pro-apoptotic, cell cycle arresting, autophagy, antiangiogenic and antimetastatic effects of luteolin are considered in diverse experimental models of different cancer types. In addition, combinatorial activities of luteolin with conventional anticancer drugs that are currently used in clinical settings are also discussed.

## 2. Chemistry Associated with Luteolin

**Figure 1:**
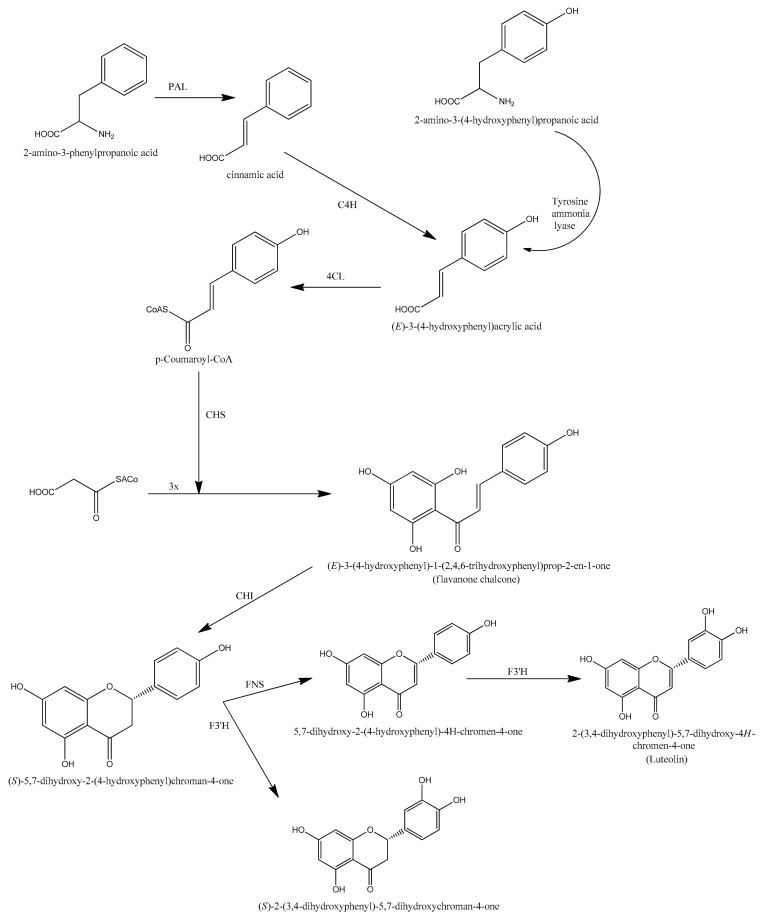
Biosynthetic routes for luteolin formation.

**Figure 2:**
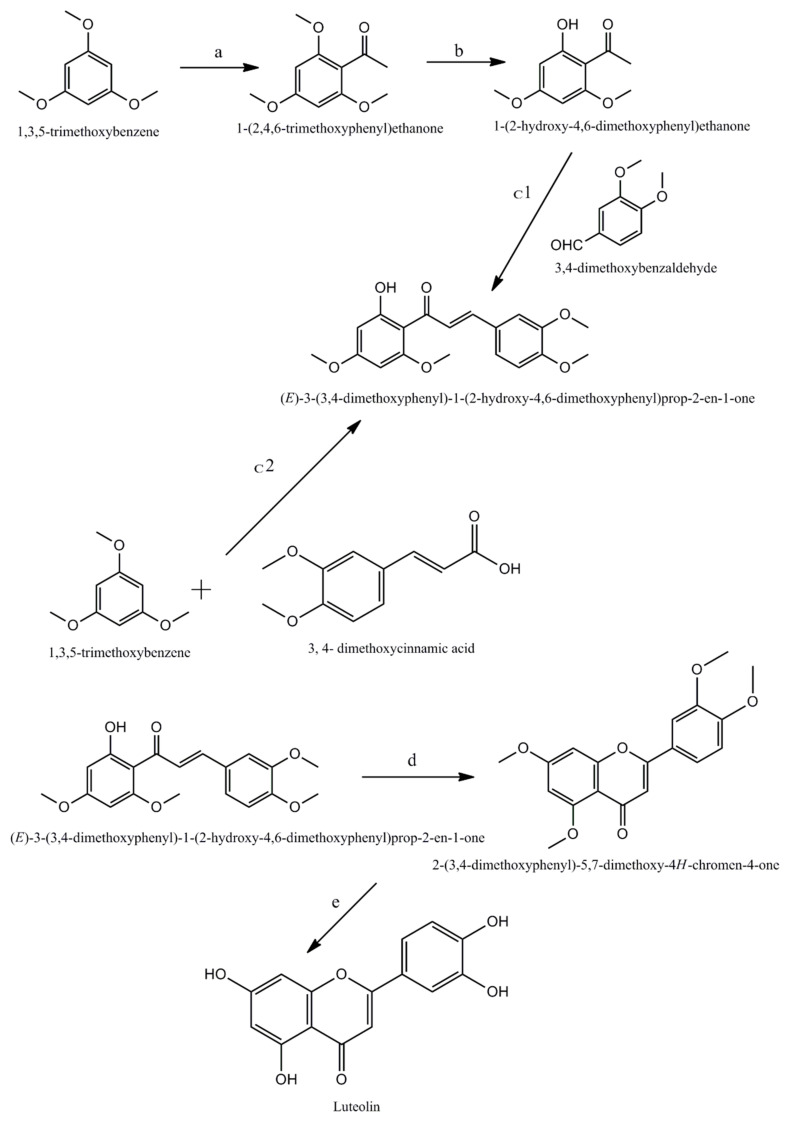
Chemical Synthesis of luteolin. a, b, c1, c2, d, and e represent the essential conditions for the completion of reaction: (a) BF3–Et2O, EtOAc, r.t., 2 h, 93%; (b) BCl3, CH2Cl2, 0 °C, r.t., 2 h, 87%; (c1) KOH, r.t., 72 h, 83%; (c2) BF3–Et2O, 100 °C, 6 h, 45%; (d) DMSO, I2, 130 °C, 4 h, 80%; (e) pyridine HCl, 180 °C, 6.5 h, 88%.

Chemically luteolin is a 3′, 4′, 5, 7 tetra hydroxyl flavonoid composed of a C6-C3-C6 carbon skeleton with two benzene rings linked by a heterocyclic ring, and having a yellow crystalline appearance [15]. It is naturally found in many plants, fruits, and vegetables. Three enzymes, namely phenylalanine ammonia lyase (PAL), cinnamate 4-hydroxylase (4CH), and 4-coumaroyl CoA ligase (4CL), use the phenylpropanoid route to biosynthesize luteolin in plants (Figure 1). Luteolin can be extracted from plants with the help of modest extraction techniques [16], but only a small amount of luteolin is available for biological studies because it is difficult to obtain. In 2014, Ji Zhang et al. disclosed a new green and convenient approach for the synthesis of luteolin using 1,3,5-trimethoxybenzene via Friedel–Crafts acylation, demethylation selectively, Claisen–Schmidt condensation with 3,4-dimethoxybenzaldehyde [17]. It was also prepared by using 1,3,5-trimethoxybenzene and 3,4-dimethoxycinnamic acid as starting materials in the presence of excess of BF_3_–Et_2_O through one-pot (Figure 2) reaction.

**Figure 3:**
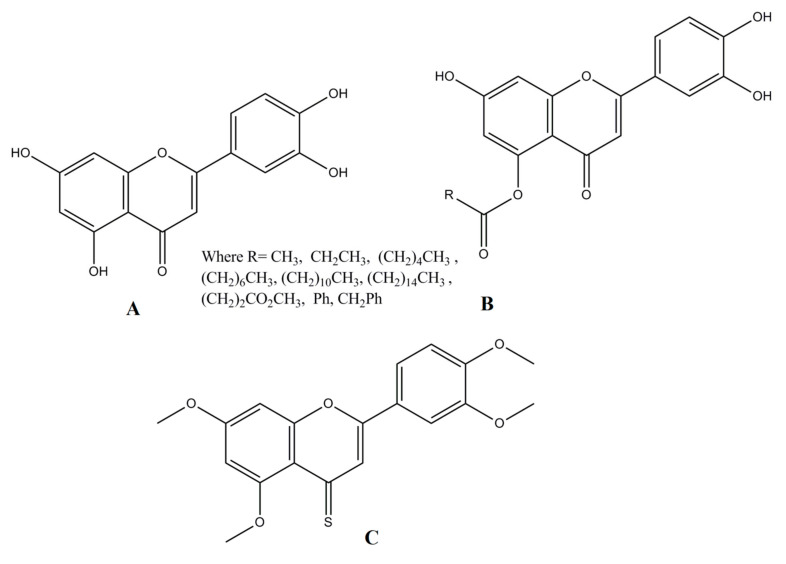
Luteolin and its derivatives. (**A**) 2-(3,4-dihydroxyphenyl)-5,7-dihydroxychromen-4-one (**B**) Monoacylated luteolin (**C**) 2-(3,4-dihydroxyphenyl)-5,7-dihydroxy-4H-chromene-4-thione.

The primary cause of flavonoids’ biological activity is the presence of their hydroxyl groups. Luteolin has four hydroxyl groups at the locations of C5, C7, C3′, and C4′ that provide potent anti-oxidative properties, while the double bond between C2 and C3 is responsible for its efficient biocidal activity. It is effective against microorganisms, due to the carbonyl oxygen present at C4 locations (Zhao et al. 2021). Biologically, potent derivatives of luteolin have also been reported by various authors. In a recent report, Stephen Lo et al. [18] reported mono-acylated luteolin derivatives (Figure 3) with enhanced antiproliferative activities against HCT116 and MDA-MB-231 cancer cell lines. Similarly in another report, Divyashree Ravishankar et al. reported an antiangiogenic [19] 4-thiomethoxy derivative of luteolin.

## 3. Absorption and Metabolism of Luteolin

Luteolin, a naturally occurring flavone, is majorly available in the form of aglycone or glycosides. Following intestinal absorption, the majority of luteolin is conjugated, leaving behind a small proportion of free luteolin in the body. Hence, the bioactivity of luteolin can primarily be attributed to its metabolites [20] catalyzed by UDP-glucuronosyltransferases (UGTs) and catechol-O-methyltransferases (COMTs) [21]. One report suggested glucuronidation and methylation to be two key pathways mediated via the interplay of UGTs and COMTs involved in the metabolic disposition of luteolin [21]. Another group confirmed that luteolin glucuronides, especially luteolin-3’-O-glucuronide comprise the active compound of luteolin, which exhibits its anti-inflammatory effect in vivo [20]. This was supported by another investigation that examined the intestinal absorption of luteolin and luteolin 7-O-beta-glucoside in rats by HPLC and demonstrated that luteolin was converted to glucuronides during its passing through the intestinal mucosa. Moreover, luteolin 7-O-beta-glucoside upon hydrolysis into luteolin was absorbed. Hence, the major forms of luteolins found in the rat plasma included free luteolin, its conjugates and methylated conjugates. The presence of free luteolin suggested that some luteolin could escape the intestinal conjugation or the hepatic sulfation/methylation. This HPLC analysis validated the presence of free luteolin and its monoglucuronide in human serum after ingestion of luteolin [22]. This observation was supported by another study which showed that luteolin, luteolin monoglucoside, and luteolin monoglucuronide may circulate in humans [23]. In another study, the oral administration of luteolin glucosides and luteolin aglycone to rats resulted in the detection of luteolin glucuronides in plasma and organs [24]. An HPLC-MS/MS analysis of luteolin and its active metabolites (diosmetin, chrysoeriol, and luteolin-7-O-glucuronide) in rat plasma suggested that the enzymatic activity of UDP-glucuronosyltransferases had a great influence on its pharmacological activity or toxicity [25]. In addition, extensive glucuronidation by uridine diphosphate-glucuronosyltransferases 1As (UGT1As) results in poor bioavailability of luteolin, which limits its clinical application. Hence, a recent report indicated that resveratrol (RES), an inhibitor of UGT1A1 and UGT1A9, had a significant effect on the enhanced bioavailability of luteolin by reducing the major glucuronidation metabolite in rats, which provides a check-point for manipulation of the LUT/RES axis in liver diseases [26].

## 4. Mechanistic Insight into the Anticancer Activity of Luteolin

### 4.1. Apoptotic and Cell Cycle Arrest Mechanisms of Luteolin

**Figure 4:**
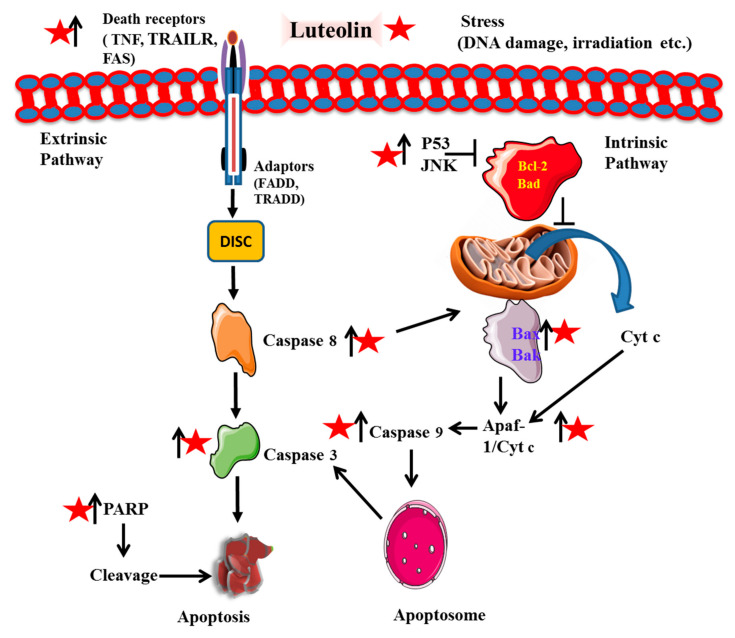
Intervention of luteolin into apoptotic mechanisms of cancer cells. Luteolin is represented by a red star, whereas arrows designate up (↑) and downregulation (↓) of the molecules. It modulates the expression of anti-apoptotic factor (Bcl-2), and apoptotic (Bax, Bak, Cyt c, Caspases and Apaf) for the progression of natural cell death.

Apoptosis induction (natural cell death) and cell cycle arrest are known to be promising drug targets opted for by a variety of chemotherapeutics and phytochemicals. Luteolin is found to possess both extrinsic as well as intrinsic mechanisms of apoptotic cell death in cancer (Figure 4). For instance, Wang et al., 2018 evaluated PARP (poly (ADP-ribose) polymerase) cleavage and upregulation of Fas and Fas ligand (FasL) along with increased levels of caspases-8 and -3 [27]. For instance, a recent study has shown that administration of 25 μM luteolin significantly reduces cell viability by inducing apoptosis in p53-deficient cell lines by significantly increasing the cell proportion at the sub-G_0_/G_1_-phase of cell cycle and decreasing the cell proportion at S-phase [28]. It increased p53 phosphorylation and p53-targeted downstream gene expression, initiating apoptosis and cell cycle arrest [29]. Suppression of CDK2 activity in cancerous cells HT-29 and OCM-1 cells is related to G_1_ cell cycle arrest [30]. Dose- and time-dependent effects of luteolin were observed on the cytotoxicity of human colon cancerous LoVo cells with IC50 of 66.70 µmol/L (24 h) and 30.47 µmol/L (in 72 h). Similar results were observed in the human colon HCT-15 cell line [31]. This was due to the cell cycle arrest at G_2_/M phase that ultimately resulted in cellular apoptosis [32]. Luteolin leads to the inactivation of essential cell-cycle proteins such as cyclin B, CDC2 (cell division control), procaspase-9 in mice models and upregulated cyclin A, APAF-1, cytochrome C, caspase-9 and-3, and cyclin-dependent kinases (CDK) 2 [32]. These proteins play a very essential role in cell division and cell cycle progression. It effectively suppresses the expression levels of p-STAT3 (signal transducer and activator of transcription), p-Akt, p-EGFR (epidermal growth factor receptor), and p-Erk1/2 (extracellular signal-regulated kinase) in cancerous cell lines [30]. Inhibiting CDC1/CDK2 and cyclin B1/CDC2 proteins successfully arrested the cell at the G_2_/M transition. Cytochrome c and APAF 1 (apoptotic protease activating factor1) activates caspase recruitment domain (CARD) which in turn activates caspase-9 to form apoptotic bodies. This initiates caspase-3 and other caspase cascade reactions resulting in apoptosis of the cell [33]. Similar effects were also observed in esophageal cancer Eca109 cell line and A172 and U-373MG, human glioblastoma cell lines [34,35]. The apoptosis of breast cancer cell line MDA-MB-231 was observed to be induced by downregulating human telomerase reverse transcriptase protein (hTERT). It inhibited the phosphorylation of NF-κB (nuclear factor kappa B) inhibitor α and its subsequent target gene c-Myc (master regulator of cell cycle) followed by the suppression of hTERT [36]. Treatment of cancerous cells with luteolin significantly decreased BCL-2 (B cell leukemia/lymphoma 2) and VEGF (vascular endothelial growth factor) expression, while increasing the expression of BAX protein (Bcl-2-associated X protein). This signaling initiated the mitochondrial-modulated function to cause cell death [37]. A recent study has shown luteolin to suppress tumor proliferation by inducing apoptosis through MAPK pathway (mitogen-activated protein kinase) activation. LIM domain kinase (LIMK) 1 protein and its associated proteins (such as Ki-67, p-LIMK, p-cofilin), which are highly expressed in the lung cancer cell line, are significantly inhibited by luteolin [38]. It also induced potential mitochondrial membrane collapse, thereby leading to cytochrome c release, and an increased expression of BAX by inhibiting the expression of Bcl-2. Furthermore, it also enhanced the expression of death receptors DR5, which activated caspase-8/-9/-3 cascades in MCF-7 cells [39]. These overall mechanisms also significantly decreased the tumor size and weight, thereby leading to cell-cycle arrest and apoptosis [32]. Furthermore, luteolin inhibited the proliferation of human colon adenocarcinoma cell line HT29 by increasing the expression of Caspase-1, Gasdermin D and IL-1β, members of pyroptosis, a form of cell death [40].

### 4.2. Autophagy- Inducing Mechanism of Luteolin

Autophagy is a process that degrades cells and removes toxic substances from cells that are under stress, and functions as a self-degradation system [41]. The autophagy process is classified into different types i.e., micro, macro and chaperone-mediated autophagy that transmits to the lysosome. Macroautophagy is a metabolic process that wraps protein cells to form autophagosomes with a bilayer membrane, the membrane fuses with the lysosomal membrane and degrades the wrapped protein by hydrolyzing [42]. Luteolin affects various pathways, i.e., it is involved in autophagy that includes nucleation and elongation that prevents the progression of cancer. Luteolin attenuates Wnt signaling (Wingless-related integration site) pathway for the upregulation of fizzled class receptor to downgrade cancer cells. Beclin1 plays an important role in autophagy, a process involved in cell survival that increases during cell stress and decreases over the cell cycle. The Beclin1 regulates autophagy during the initiation step that suppresses tumors and downregulates the Beclin1 expression in cells. Luteolin affects the ER chaperone binding and activates stress sensors and induces autophagy [43]. The Beclin1 promotes protein light chain formulation that effects elongation steps through the downregulation of light chains. Autophagy can also help in the survival of cells in cancer cells with Beclin1 downregulation [44].

**Figure 5:**
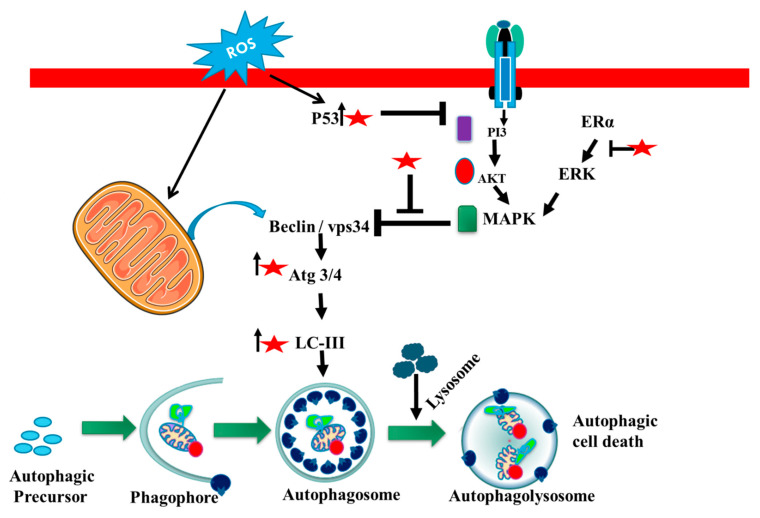
The role of luteolin in regulation of autophagy. Luteolin is found to modulate the expression of autophagic molecules including MAPK, Beclin1, and LC3 to initiate autophagy in cancer. Luteolin is represented by a red star, whereas arrows designate up (↑) and blockage (┴) of the molecules.

The high amount of luteolin may cause lethal autophagy in lung cancer, proving induction of caspase-dependent programmed cell death. The major role of luteolin was reported to increase LC3 (microtubule-associated protein 1A/1B-light chain 3) puncta and autophagy flux by activating caspases and beclin1 [35,45]. Luteolin also inhibited cancer cell development via the Wnt β-catenin pathway, and may clearly halt the cell cycle by decreasing Akt-phosphorylation, which further leads to dephosphorylation and triggers GSK-3 (glycogen synthase kinase). Upon activation of GSK-3, the level of cyclin D1 phosphorylation rises at Thr-286, with proteasomal destruction [46]. During sensitization of cancer cells luteolin makes a significant impression on cleaving caspase and inhibits cancer cells by stimulating autophagy. Luteolin, upon activation of MAPK activation, decreases the proliferation that leads to the downregulation of P62, leading to autophagy and induces FADD (Fas-associated death domain)-mediated apoptosis [47]. Therefore, luteolin in cancer therapy could be beneficial to reduce tumor cell survival and proliferation via autophagy regulation (Figure 5).

### 4.3. Antiangiogenic and Antimetastatic Action of Luteolin

It is well established that angiogenesis plays a prominent role in the occurrence, invasion and metastasis of tumors. Significant findings on the antiangiogenic and antimetastatic properties of luteolin have yielded positive results [48,49,50]. Studies have demonstrated that luteolin inhibits breast cancer invasion via inhibiting VEGF production and the receptor activity, and also it decreases the expression of markers for epithelial–mesenchymal transition and inclination towards metastasis. Some studies have also demonstrated that luteolin suppresses angiogenesis by stabilizing hyaluronic acid, an anti-angiogenic barrier. It has been observed that if hyaluronic acid is catalyzed by hyaluronidase, a cascade of events results in neo-vascularization. Luteolin, on the other hand, is found to be a potent inhibitor of hyaluronidase in maintaining the barriers of neovasculature [5,50,51,52,53].

The ability to invade surrounding tissue and migrate from the primary site is an important characteristic of cancer. Varied studies have also shown that luteolin blocks the expression of MMPs, pro-inflammatory cytokines such as TNF-α (tumor necrosis factor), IL-6 (interleukin), IL-1, NF-κB, and endothelial migration, the factors involved in tumor progression and metastasis [54,55,56]. It has been observed that luteolin acts on tumor-associated macrophage (TAM) and other associated immune cells which releases chemokines, e.g., C-X-C chemokine receptor type 4 (CXCR4, a growth factor involved in the metastasis of cancer [54,55,57].

Luteolin not only downregulates the expression of anoctamin 1, a calcium-activated chloride channel, but also inhibit its functional activity that leads to inhibition of cell proliferation, migration and invasion in prostate cancer cells [58]. Furthermore, epithelial to mesenchymal transition (EMT) plays an important role in cancer metastasis. Luteolin causes repression of EMT via targeting several associated transcription factors, markers and signaling pathways [5]. Additionally, luteolin treatment resulted in the loss of cell–cell adhesion and an increased cell invasion via increased expression of E-cadherin by inhibiting mdm2 through the AKT pathway in prostate cancer PC3 cells [59].

**Figure 6:**
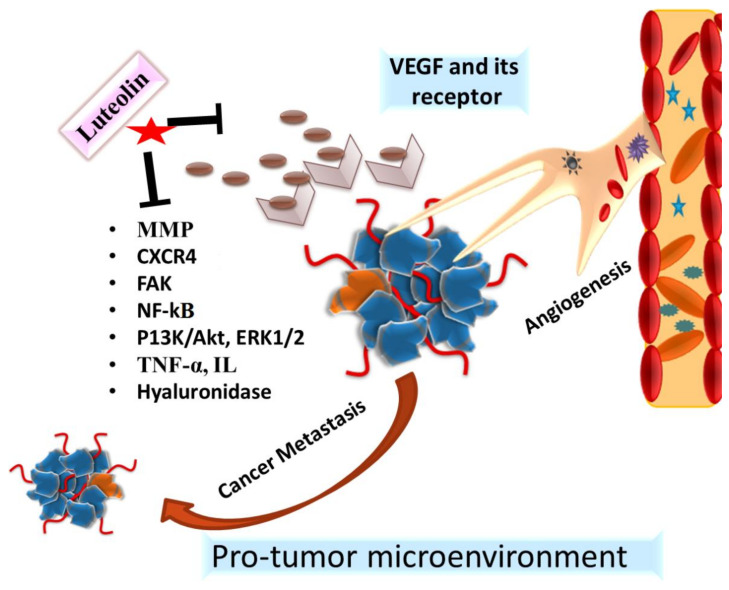
Molecular mechanisms of antiangiogenic and antimetastatic activities of luteolin. It regulates the expression of angiogenic (VEGF/VEGFR), and metastatic proteins (MMPs, CXCR4, FAK, PI3K/AKT, mTOR, ERK) to inhibit neo-asculature and cancer migration respectively.

Studies have shown that crucial signal transduction pathways involved in cancer cell metastasis and progression are blocked by luteolin, for example, EGFR activation. Luteolin has shown to block the EGFR-signaling pathway, thereby reducing cell invasion and metastasis. Studies have demonstrated the inhibitory potential of luteolin on focal adhesion kinase (FAK) activity in cancer thereby halting cell invasion [60,61]. Luteolin has been found to be effective and exert an inhibitory effect on the proliferation, migration, and invasion of different cancers via acting on and altering the PI3K/AKT, mTOR (the mammalian target of rapamycin), ERK, and p38 signaling pathways and their associated molecules [62] (Figure 6). Despite the availability of many in vitro and in vivo studies, not many clinical studies have been conducted to explore the beneficial properties of luteolin [6,63]. The need of the hour is to explore dedicated model studies on the antimetastatic and antiangiogenic properties of luteolin, along with the delineation of cancer inhibitory pathways.

### 4.4. Immunomodulatory Mechanisms of Luteolin

**Figure 7:**
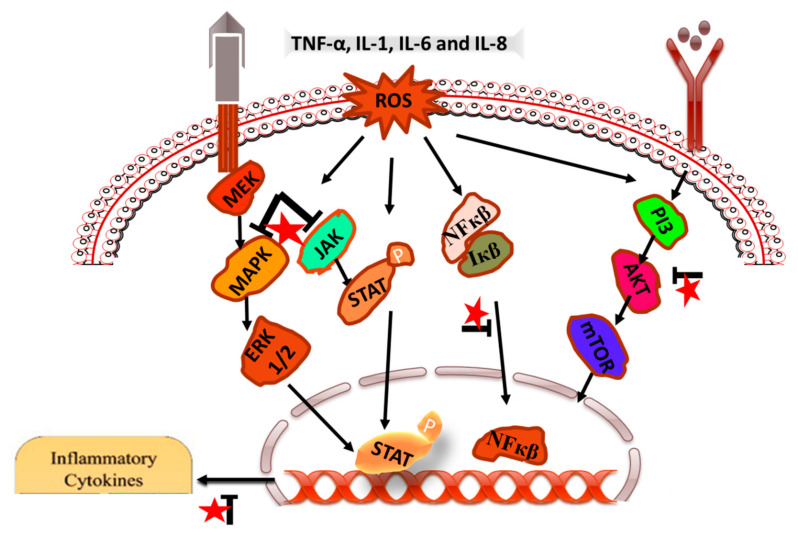
Different pathways inhibited and modulated by luteolin for anti-inflammatory activity. For instance, downregulation of NF-κB, Akt, MAPK, ERK, STAT, (IL)−1β, IL-6, IL-8, and TNF-α, is found to be initiated by luteolin to inhibit inflammatory microenvironment. Luteolin is represented by a red star, whereas arrow designate blockage (┴) of the molecules.

Inflammatory reaction occurs in response to harmful a stimulus such as injury, stress and microbial invasion. It is carried out by immune and non-immune cells in a well-defined coordinated manner in order to maintain homeostasis, through activation of signaling pathways. Numerous flavonoids have been reported for their anti-inflammatory activity and are under clinical trials to be used for drug development. Luteolin is one of the flavonoids having anti-inflammatory mechanism at micromolar concentrations and acting as a promising compound for further development [64]. Luteolin is nontoxic in nature, but not granted generally recognized as safe status by USFDA. The anti-inflammatory mechanisms of the action of luteolin relates to its ability to inhibit NO production, nitric oxide synthase (iNOS) expression, and ROS production. Furthermore, luteolin activates antioxidant enzymes, scavenge reactive oxygen species (ROS), promotes leukotriene production, adhesion- molecule membrane-binding-inhibition, hyaluronidase and elastase activity, vascular-permeability reduction and cell membrane-fluid modulation, mast cells-stabilization inhibition, proinflammatory cytokine-expression suppression, NF-κB pathway, Akt and the mitogen-activated protein kinase (MAPK)-pathway inhibition (Figure 7).

Coordinated activation of a signaling pathway for inflammatory response is crucial to maintain the balance between pro-inflammatory and anti-inflammatory mediators [65]. By regulating the inflammatory mediators and cytokine production, luteolin has been shown to exert its anti-inflammatory effects. Acute and chronic inflammation is modulated by cytokine by acting as key modulator [66]. The level of IL-10 (anti-inflammatory) increases by the luteolin, through the interleukin (IL)−1β, IL-2, IL-6, IL-8, IL-12, IL-17, TNF-α, interferon (IFN)-β, and granulocyte-macrophage colony-stimulating factor inhibition. In addition to this, luteolin also inhibits the chemokines, along with prostaglandin and leukotriene which play a crucial role in immune cell migration [67]. Luteolin exerts its anti-inflammatory activity through the inhibition of iNOS (inducible nitric oxide synthase) function, iNOS expression, and NO production, as NO is a labile radical entity and ROS is regulated by luteolin [68]. It has been reported that luteolin acts as an activator of antioxidants and ROS scavenger [64]. Lactate dehydrogenase (LDH) production was decreased and superoxide dismutase (SOD) activity with intracellular level of glutathione (GSH) was found to be elevated in endothelial cells after the luteolin attenuation of TNF-α-induced intracellular ROS generation [69]. Luteolin has been found to suppress the phosphatidylinositide 3-kinases (PI3K)-AKT-NF-κB-extracellular signal-regulated protein kinases 1 and 2 (ERK1/2) pathway, which leads to a decline in ROS levels in the case of zinc-induced apoptosis of human neuroblastoma SH-SY5Y cells [70].

NF-κB transcription factor plays an important role in pro-inflammatory genes expression and its inhibition mediates the anti-inflammatory activity of luteolin. NF-κB selective stimulation leads to IκB kinase (IKK) complex-mediated IκB protein degradation via phosphorylation, which further results in nuclear translocation of NF-κB and induces the transcription of target genes. Natural compounds such as luteolin inhibit the NF-κB signaling pathway, which plays an important role in the generation of inflammation [71,72]. Mitogen-activated protein kinases (MAPK) and AP-1 signaling were also modulated by the luteolin. It was reported that in SW982 cells, luteolin affects the MAPK pathway through IL-1β-induced c-Jun N-terminal kinase (JNK) suppression and p38 kinase activation. Additionally, IL-1β-induced nuclear translocation of AP-1 inhibition was also observed [73]. ROS-scavenging activity of luteolin has also been reported through the inhibition of the MAPK pathway, which is activated by ROS [64]. It has been reported that ROS-induced activation of the MAPK pathway is attenuated by luteolin [74]. In addition to this, ERK1/2 phosphorylation is significantly enhanced by luteolin. In a ROS-activated MAPK pathway in Sprague-Dawley rats and H9c2 cells, p-p38 MAPK and p-JNK (c-Jun N-terminal kinases) levels were reported to be decreased [74]. Luteolin was also found to decrease the STAT-binding activity and STAT1 phosphorylation, which further decreases the IRF-1 (interferon regulatory factor 1) basal levels, which is a transcriptional factor regulating proinflammatory cytokine expression [75]. Therefore, it can be concluded that the luteolin exerts its anti-inflammatory activity through different mechanisms, and activity varies with the signaling pathway.

### 4.5. microRNA (miRNA) Modulations by Luteolin in Cancer

MicroRNAs (miRNAs) are endogenous, 18–22 nucleotide long noncoding RNAs that regulate gene expression post-transcriptionally by either translational repression or degradation of target mRNA [76,77]. Recent research has shown that modification of the expression of miRNAs that play significant roles in the biology of the tumor, including cell proliferation, and metastasis can reverse the cancer phenotye [78].

Research to date suggests that phytochemicals can drastically alter a number of miRNAs linked to cancer, hence preventing the onset and progression of cancer [79]. Luteolin-mediated control of miRNAs is an intriguing and growing field. It is exciting to note that a systematic and sequential accumulation of information has begun to illuminate the complex control of microRNAs by luteolin in various malignancies. Together, advanced data will allow us to create a more in-depth comprehension of how luteolin regulates signaling pathways and miRNAs on multiple levels in various malignancies. According to research involving several cancer cell lines, miR-34, a crucial tumor suppressor gene, was increased after treatment of luteolin [80,81,82,83]. Along with miR-34, studies have shown that luteolin treatment of cancer cells upregulated a number of other tumor suppressors, including miR-9, miR-7-1-3p, miR-181a, miR-5703, miR-195/215, miR-630, let-7c, miR-139, miR-221, miR- 98, miR-107, miR-422a, miR-6809-5p, miR-224, miR-139-5p, miR- 181a, miR-124-3p miR-384, while downregulating a number of oncogenes, such as miR-340, miR-301, miR-155, miR-21 and miR-224 [84].

Furthermore, in prostate cancer cells, luteolin administration reduced cell growth and caused apoptosis by downregulating miR 301 and inducing the production of death effector domain-containing protein 2 (DEDD2), a pro-apoptotic molecule [85]. According to Zhou et al., luteolin increased the expression of miR 34 in gastric cancer cells, and miR 34 overexpression made cells more susceptible to luteolin [83]. When luteolin was given at a high dose (200 mg/kg) to a non-small cell lung cancer (NSCLC) animal model by microarray analysis, miR-34a was found to be highly expressed [81]. By increasing miR-34a-5p and targeting MDM4, luteolin also reduced carcinogenesis and triggered death in non-small cell lung cancer cells [81]. Luteolin modulates PTN via the expression of miR-384 to cause anticancer effects on colorectal cancer cells (Yao et al., 2019b). Luteolin exposes cells of pancreatic ductal adenocarcinoma, attenuates cell proliferation and enhances the anti-proliferative effect of TRAIL on cancer cells by downregulation of miR-301-3p [86]. Luteolin could significantly inhibit NOTCH signaling by regulating various miRNAs such as the upregulation of miR-121a, miR-34a, miR-224, miR-246, miR-139-5p and downregulation of miR-155 involved in tumor development and progression in breast cancer [87]. Furthermore, in the breast cancer cell line MCF-7, luteolin considerably increased miR-16 and miR-34a expression while significantly decreasing miR-21 expression and resulted in decreased cell viability, caused a large buildup of apoptotic cells in the sub-G1 and G0/G1 cell cycle phases, and triggered apoptosis by upregulating BAX, a pro-apoptotic, and downregulating Bcl-2, an anti-apoptotic protein [88]. Additionally, luteolin stimulated miRNA-203 expression and targeted Ras and Raf expression in breast cancer cells (MDA-MB-453 and MCF7). Additionally, it was discovered that breast cancer cells lacking miR-203 had increased levels of p-MEK and p-ERK, which is also found to be increased in renal cell carcinoma [89]. This indicates the role of luteolin in inhibiting cancer progression via miRNA [90]. In gastric cancer cells, luteolin administration dramatically elevated the tumor-suppressor miR-34a, miR-139, miR-107 and miR-422a levels, while considerably decreasing the oncogene miR-155, miR-340, miR-21 and miR-224 levels [82].

One of the studies showed that luteolin treatment resulted in the overexpression of miR-7-1-3p that leads to inhibition of autophagy and also apoptosis induction [91]. Additionally, Yao et al. examined the relationship between miRNAs and luteolin in glioma cells. The findings showed that luteolin treatment of glioma cells dramatically enhanced miR-124-3p expression, increasing cellular cytotoxicity. By triggering apoptosis and autophagy through the activation of MAPK in glioma, luteolin may be able to inhibit the growth of tumors. In U251 cells and LN229 cells, miR-124-3p overexpression may greatly increase the amount of cleaved caspase-3. Following luteolin administration, cleaved poly (ADP-ribose) polymerase, caspase-3 and caspase-8 levels significantly increased, and these are involved in apoptosis via an extrinsic pathway. Furthermore, p38, JNK, and ERK could all be activated and phosphorylated by luteolin [92].

**Figure 8:**
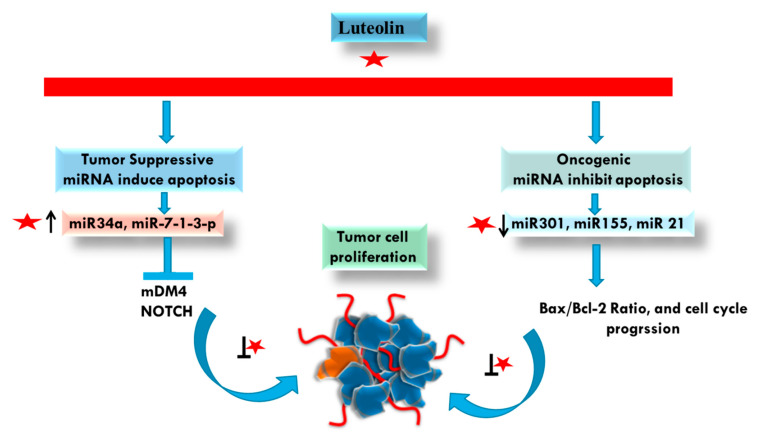
Regulation of cancer progression by luteolin through affecting different miRNAs. Luteolin can increase and decrease the expression of tumor suppressive (MiR34α, miR-1-3-p) and oncogenic (miR301, MiR155, miR21) miRNA, respectively. Luteolin is represented by a red star, whereas arrows designate up (↑), downregulation (↓) and blockage (┴) of the molecules.

The anticancer properties of luteolin may be influenced by miRNA-related processes. The downregulation of oncogenes and/or activation of tumor suppressors, which can influence proliferation, migration, invasion and apoptosis in cancer cells, may be some of these methods. These results add credence to the idea that luteolin, a substance derived from natural products, may be a treatment for cancer (Figure 8). To establish the practical applicability of these findings and to investigate which miRNAs are crucial for the molecular activities of luteolin in cancer, more research, particularly clinical trials, is required.

## 5. Synergistic Effects of Luteolin with Conventional Anti-Cancer Drugs

Treatment of cancerous cells with luteolin has proven to be effective; however, there have been many studies highlighting the synergistic effects of luteolin with other natural/synthetic drugs, showing new possible treatment options. A recent study analyzed the combinatorial treatment of luteolin with oxaliplatin, and observed a significant decrease in the expression of p21 protein in p53^+/+^ HCT116 cells. The results showed synergistic effects as compared to treatment with oxaliplatin or luteolin alone [28]. Luteolin with hesperidin also effectively downregulated miR21 expression levels while upregulating miR-16 expression levels in the breast cancerous-cell line MCF-7 [88]. Luteolin-complexed nanoparticles efficiently decreased the expression levels of mRNA of downstream gene Nrf2 to a greater extent, as compared to luteolin alone [30]. Synergism of luteolin with sulforaphane was observed at a molecular level. Reduction in expression levels of proteins involved in the NF-κB pathway and STAT3 activation was observed [93]. Similar effects were observed with a combination of celecoxib and luteolin in breast cancer cell lines such as MDA-MB-231. This combination demonstrated a greater increase in cell apoptosis, which was attributed to decreased levels of p-Akt [94,95]. Similarly, combination treatment with luteolin and quercetin on nicotine-treated MDA-MB-231 cells has been shown to enhance antiproliferative effects by downregulating nicotinic acetylcholine receptors [96]. Recently, luteolin with oxaliplatin was studied, to suppress the proliferation of gastric cancerous cells SGC-7901 through modulation of the Cyt C/caspase pathway; this increased the levels of cyclin D1, arresting the progression of cells at G_0_/G_1_ phase [49]. The combination of luteolin and lapatinib synergistically inhibited the expression of ERBB1, ERBB2 mRNA, and the phosphorylation level of Akt, ERK1/2 in breast cancer BT474 cells [97]. Cell migration and the invasion of glioblastoma SNB19 cells as well as glioblastoma stem cells, were significantly reduced upon treatment with luteolin and silibinin. The combination induced apoptosis, by inhibiting cell cycle proteins of intrinsic and extrinsic pathways such as PKC-α, XIAP, and iNOS [98]. Luteolin, along with 5-fluorouracil, was administered to hepatocellular carcinoma cells (HepG2 and Bel7402), showing enhanced expression of Bax/Bcl-2 ratio, p53 protein, and induction of apoptosis through PARP cleavage [99]. Similarly, the administration of luteolin and 5-FU demonstrated apoptosis induction via increased levels of p53, p21, and caspase 3 in the solid Ehrlich carcinoma mice model [100]. Additionally, the combined treatment of luteolin and oxaliplatin significantly increased apoptosis in the colorectal carcinoma xenograft-mouse model by elevating the expression of cleaved PARP and p53 via inhibition of the AMPK pathway [101]. Administration of polyphenols such as (-)-epigallocatechin-3-gallate with luteolin has been observed to synergistically inhibit TGF-β through RhoA and ERK inhibition pathways, and to decrease the serum levels of HGF and VEGF in prostate cancer cells [102]. A combination of the Cisplatin drug with luteolin synergistically inhibited the migration and invasion of the ovarian cancer cell line CAOV3/DDP in a dose-dependent manner [103]. A recent study has highlighted the significant positive effects of a combination of luteolin with three polyphenols i.e., quercetin, apigenin, and p-coumaric acid, on the antiproliferative activities of MCF-7 breast cancer cell lines of up to 90% [104]. Such studies signify that the synergistic effects of luteolin with other natural/synthetic drugs may prove to be more beneficial than luteolin alone.

The results obtained for the synergistic effects of luteolin and conventional chemotherapeutics such as 5-fluorouracil and cisplatin, clearly show that in the future, treatment regimens combining this natural flavone with anticancer drugs might be developed to lower the efficient doses of chemotherapeutics and thereby mitigate also adverse side effects caused by these drugs. Moreover, the presence of luteolin might even provide some protective effects against chemotherapeutics-induced toxicities, such as doxorubicin-induced cardiotoxicity [105] or cisplatin-induced nephrotoxicity [106]. However, more in vivo studies with the initiation of clinical trials are urgently needed to further evaluate these attractive insights.

## 6. Insight into the Nanodelivery of Luteolin in Cancer

Poor solubility of flavonoids has always been a daunting task for scientists, being widely researched to explore their hidden therapeutic mechanisms. As discussed above, luteolin is known to inhibit tumorigenesis in a diverse range of cancers by inhibiting the viability, migration, angiogenesis and invasion [11,12,107,108,109,110,111,112,113,114,115]. However, the combination of protein and flavonoids can ameliorate the problems of poor solubility and stability of flavonoids for better utilization. In one of the recent studies, a soy protein isolate pretreated by ultrasonication was selected as the embedding wall material, which was combined with luteolin to form a soy protein isolate (SPI)-luteolin nanodelivery system. This SPI delivery system increased the luteolin release rate and the utilization of fat-soluble active substances [116]. Another recent report investigated the preparation and evaluation of a non-invasive intranasal luteolin delivery for the management of cognitive dysfunction in Alzheimer’s disease (AD), using novel chitosan-decorated nanoparticles. The prepared nanoparticles proved to be a promising safe, effective, and non-invasive nanodelivery system that improved luteolin delivery which in turn enhanced cognitive function in AD patients [117]. Luteolin-loaded nanovesicles (LT-NVs) prepared by a solvent evaporation method using cholesterol, phosphatidylcholine, span 60, and labrasol in different compositions revealed enhanced drug release as well as permeation profile. The enhanced permeation from LT-NVs was achieved due to the enhanced solubility of luteolin in the presence of the surfactant, concluding that LT-NVs are a natural alternative to the synthetic drug in the treatment of lung cancer [118]. As such sporadic reports have been documented focusing on nanodelivery techniques of this flavone in cancer, exploring this area holds potential for therapeutic targeting of carcinogenesis by effective delivery of luteolin, an effective anti-cancer agent.

## 7. Safety Studies Related to Administration of Luteolin

As a dietary phytochemical, luteolin is considered to be generally safe. This assumption has been confirmed also in several specific studies. Xiong et al. showed that this flavone, administered intraperitoneally at 100 mg/kg, displayed no obvious liver or kidney toxicity in male mice, suggesting a good safety profile. In this work, the LD_50_ for luteolin was calculated to be 460 mg/kg [119]. In rats, the intraperitoneal and oral LD_50_ values for luteolin were estimated to be 411 mg/kg and >5000 mg/kg, respectively [120]. De Leo et al. described the favorable safety of luteolin also in zebrafish larvae [121]. However, the safety profile of luteolin in humans has still remained unclear, and definitely needs to be evaluated in further clinical trials. Table 1, Table 2, Table 3 and Table 4 represent an overview of the anti-cancer potential of luteolin in various in vitro and in vivo models.

## 8. Conclusions

The data discussed in the current review article clearly show that luteolin might be considered a potent molecular lead for the further design of anticancer agents. This natural flavone alleviates inflammation, inhibits proliferation, migration and invasion of cancer cells, and promotes the death of malignant cells through different mechanisms (apoptosis, autophagy). Luteolins has also been shown to potentiate the anticancer activity of conventional chemotherapy, when administered together. Therefore, further studies with luteolin to improve its targeted delivery to cancer tissues by nanotechnological approaches, as well as establishing its safety profile in humans, are urgently needed, to utilize more intensively the anticancer potential of this attractive phytochemical. Different strategies, including the application of structure–activity relationship studies, might be helpful to make progress in this path.

## Figures and Tables

**Table 1:** Synergistic mechanisms of luteolin with other anticancer agents.

S. No.	Combination of Drug Molecules	Type of Cancer	Model System (Cell Lines)	Physiological Effect and Mechanism(s)	Dose	Ref.
1	luteolin and oxaliplatin	tumor	HCT116 cells	reduced the expression of p21 protein	-	[101]
2	luteolin and oxaliplatin	Tumor	gastric adenocarcinoma cell line (SGC-7901)	blocked cell progression in the G0/G1 phase and induced apoptosis; increased cyclin D1 levels	LUT (40 μM) and OXA (30 μM); 24 h	[92]
3	celecoxib and luteolin	Malignant tumors	breast cancer cells (MCF-7 and MDA-MB-231)	increased cell proliferation, cell death, apoptosis; decreased levels of Akt phosphorylation (pAkt);	10, 25, 50, 75, 100 μM for 72 h	[94]
4	quercetin and luteolin	malignant	MDA-MB-231 cell	downregulation of nicotinic acetylcholine receptors and R9-nAChR expression	0.5 μM	[122]
5	luteolin and silibinin	malignant	glioblastoma SNB19 cells and glioblastoma stem cells	prevented cell migration and invasion and induced apoptosis; targeted PKCα and iNOS	20 µM LUT and 50 µM SIL	[35]
6	apigenin and luteolin	malignant	MDA-MB231 cell	inhibited CCID, MMP1-induced calcium increase and phosphorylation of FAK essential for FAK activation; p53 signaling pathway was activated; NF-κB pathway inhibition	-	[123]
7	luteolin and cyclophosphamide	Tumor cells	human breast cancer cell	increasing Bcl-2 protein level and antioxidant activity; downregulation of Akt phosphorylation	Lut 30 mg/kg + cyclophosphamide 10 mg/kg	[40]
8	luteolin and lapatinib	-	BT474 breast cancer cells	inhibited expression of ERBB1, phosphorylation level of Akt, ERK1/2	-	[124]
9	CD55-TRAIL and luteolin	Tumor cells	colorectal cancer (CRC), HT-29 cells	displayed greater chromatin condensation, nuclear fragmentation and apoptotic body formation	CD55-TRAIL (15 MOI), luteolin (25 µM), 72 h	[125]
10	epigallocatechin-3-gallate and luteolin	-	prostate cancer cells	inhibited TGF-β and ERK inhibition pathways, decreased levels of HGF and VEGF	-	[101]
11	luteolin and paclitaxel	malignant	breast cancer cell lines	regulated Caspase 8, 3, Fas	-	[35]
12	luteolin and cisplatin	malignant	ovarian cancer, CAOV3/DDP cells	induction of apoptosis and inhibition of cell migration and invasion, downregulation of Bcl-2 expression	10–40 mg/kg, 5 days	[106]
13	luteolin and hesperidin	malignant	breast cancerous cell line MCF-7	downregulated miR21 expression levels while upregulated miR-16 expression levels, caused a significant accumulation of apoptotic cells into the G0/G1	20, 60, 100 and 140 mg/mL, for 24 h and 48 h.	[35]
14	luteolin and 5-fluorouracil		human hepatocellular carcinoma cells (HepG2 and Bel7402 cells)	enhanced bax/bcl-2 ratios and p53 expressions, and induced PARP cleavage	dose ratios (luteolin: 5-fluorouracil = 10:1, 20:1, 40:1)	[100]

**Table 2:** Apoptotic- and autophagy-inducing effects of luteolin based on in vitro studies. Arrows designate up (↑), and downregulation (↓) of the molecules.

Type of Cancer	Cell Lines	Effects	Mechanisms	Concentration	References
Osteosarcoma	MG63 and U2OS	Induces apoptosis	↓ chemoresistance to doxorubicin and cisplatin, ↓ cancer cell viability and proliferation, ↑ miR-384 level, ↓ PTN expression, ↓ PTN/b-catenin/MDR1 signaling axis, ↑ doxorubicin response in doxorubicin-resistant MG63/DOX cells, ↑ miR-384 in exosomes derived from luteolin-treated MG63 cells	0, 1, 2, 3, 4, 5 μM	[126]
Colon	HCT116	Induces apoptosis and autophagy	↓ cell migration, ↓ HIF-1α-dependent transcription, ↓ G1 and G2/M cells, ↑ cells in S phase, ↑ apoptotic frequency, ↑ necrotic cell death, ↑ LC3-II, Luteolin treatment reversed increase of CD44 and CD47	2.5–200 μM	[127]
	SW620	Induces apoptosis and autophagy	↓ viability and proliferation of cancer cells, ↑ HO-1, ↑ SOD2, ↓ Bcl-2, ↑ Bax, ↑ Cleaved caspase-3, ↑ PARP cleavage, ↑ Beclin-1, ↑ Atg5, ↑ LC3B-I/II, ↑ LC3B-I, reversal of the epithelial-mesenchymal transition, ↑ FOXO3a, ↑ apoptosis, ↑ TUNEL-positive cells, ↑ p21, ↑ phospho-ERK1/2, phospho-JNK1/2 and phospho-p38 expression	0.2, 5, 10, 20, 50, 100 μM	[47]
	HT-29, SNU-407	Induces apoptosis	↓ viability of cancer cells, ↑ Bax, ↑active caspase-9 and 3, ↓ Bcl-2, ↑ protein expression of GCLc, GSS, catalase and HO-1, ↑ DNA demethylation, ↑ mRNA expression of Nrf2	0, 5, 10, 20, 30, 40, 50, 60, 70 and 80 μM	[128]
Breast	MCF-7	Induces apoptosis	↑ Cytotoxicity for cancer cell lines, ↑ anti-breast cancer activity of L-ZnONPs was mediated by polo-like kinase 1 (PLK1) proteins (In silico studies)	2.5, 5, 10, 20, and 40 µM concentrations of luteolin, zinc oxide nanoparticles, and L-ZnONPs.	[129]
	MCF7-Tam^R^	Induces apoptosis	Cell cycle arrest at the G2/M phase, ↓ mitochondrial membrane potential, ↓ PI3K/AKT/mTOR signaling pathway, ↑ p27, ↑ cleaved-Caspase 7, 8, 9, and poly (ADP-ribose) polymerase (PARP), ↑BAX and BIM, ↓ Bcl-2, ↓ p-p85, p-AKT, and p-mTOR, ↑ MLL3 and Mono methylation of H3K4, ↑ K-Ras, H-Ras, and N-Ras mRNA	0, 10, 20, and 30 μM	[130]
	MDA-MB231	Induces apoptosis and autophagy	↓ cell migration, ↓ HIF-1α-dependent transcription, ↓ G1 and G2/M cells, ↑ cells in S phase, ↑ apoptotic frequency, ↑ necrotic cell death, ↑ LC3-II, Luteolin treatment reversed increase of CD44 and CD47	2.5–200 μM	[127]
	MDA-MB-453 and MCF-7	Induces apoptosis	↓ cells viability, ↑ apoptosis frequency, ↑ Bax, ↓ Bcl-2, ↓ Vimentin, ↓ Zeb1 ↓ N-cadherin, ↑ E-cadherin, ↑ miR-203 level, ↓ Ras/Raf/MEK/ERK signaling	0, 5, 10 and 20 μM	[131]
Lung	NCI-H1975 and NCI-H1650	Induces apoptosis	↓ proliferation of cancer cells, ↓ LIMK1 activity, ↑ cell cycle arrest at G1 phase, ↑ apoptosis frequency, ↓ cyclin D1 and ↓ cyclin D3, ↑ Bax, ↑ cleaved caspase 3, ↑ cleaved caspase-7, ↑ cleaved PARP expression, ↓ caspase-3, ↓ caspase-7, ↓ p-LIMK1/2 and p-cofilin	0, 5, 10, 20 or 40 μmol/L	[38]
	BEAS-2B, KRAS-mutant human lung cell lines H358, H460, H2122, and A549	Induces apoptosis	↓ growth and proliferation of cancer cells, ↓ MUC1-C and PD-L1, ↓ p-STAT1 or STAT3, ↑ IL-2, ↓ IFN-γ-induced PD-L1 expression	Apigenin and luteolin—0, 10, 20, 30, 40, 50 μM	[132]
Non-Small Cell Lung	A549 and NCI–H1975	Induces apoptosis	↓ cancer cell viability, ↑ apoptosis, ↑ caspase-8, caspase-3 and caspase-9, ↑ DR5 expression, ↓ mitochondrial length, ↑ Drp1 from the cytoplasm onto mitochondria, ↑p- Drp1(Serine616 residue), ↑ cytochrome c release from mitochondria, ↑ cytochrome c in the cytosolic fraction, ↓ mitochondrial cytochrome c content	0, 5, 10, 20, 30, 40 μM Luteolin + TRAIL (25 ng/mL)	[133]
	A549 and H460	Induces apoptosis	↓ proliferation of cancer cells, ↑ apoptosis frequency, ↑ P53 and P21, ↓ MDM4, ↑ Caspase 3 and 9	0, 5, 10, 20, 30, 40, 60, 80, and 100 µM	[81]
Gastric	NCI-N87 and MKN28, Hs-746T	Induces apoptosis	↓ cell proliferation, invasion, and migration of cancer cells, reversed EMT by shrinking the cytoskeleton, ↑ E-cadherin, ↓N-cadherin, ↓ vimentin, ↓ Snail, ↓ β-catenin levels, ↓ Notch1, ↓ cyclin-D1, ↓ Hes-1	0, 10, 20 and 30 μM	[134]
	MKN45, MKN28, BGC823, AGS and SGC7901	Induces apoptosis	↓ Proliferation and invasiveness of cancer cells, ↑ apoptosis frequency, ↓ MMP9, ↓ p-cMet, ↓ p-Akt, ↓ p-ERK, ↑ cleaved caspase-3 and PARP-1	0–80 μM	[135]
Pancreatic	MIAPaCa2, PANC1, BxPC3, KP4, HuPT3, PK1, PA-TU-8988T, TCCPAN2 and AsPC1	Induces apoptosis	↓ cancer cell proliferation, ↓ STAT3 activity, ↓ phospho-AMPK (Thr172), ↓ phospho-p38 MAPK (Thr180/Tyr182), ↓ phospho-STAT3 (Tyr 705), ↑ phospho-GSK3β (Ser 9), ↓ DPYD expression	25 or 50 μM	[136]
	SW1990 andAspc-1	Induces apoptosis	↓ cell proliferation, ↓ BCL-2, ↑ apoptotic frequency of cells, ↑ loss of mitochondrial membrane potential, ↑ activation of pro caspase-3 and PARP	50 μM and 100 μM	[137]
Hepatocellular	HepG2 (p53 wild type) and Hep3B (p53 null type)	Induces apoptosis	↓ cancer cell numbers, ↑ Protein levels of PARP cleaved, ↓ PCNA, ↓ catalase protein levels, ↑ mRNA levels of both Bip and spliced Xbp-1, ↑ p53 protein levels, ↑ p21 gene expression↓ TAp63 mRNA levels, ↑ LC3-II, ↓ p62	0, 5, 10 μM	[138]
	SK-Hep-1 and AML12	Induces apoptosis	↓viability of cancer cells, ↑ apoptotic cell population, ↑ sub-G1 population, ↑ cleaved-caspase 8, -9 and -3, cleaved-PARP, ↓ XIAP, ↓ Mcl-1, ↓ cleaved Bid, ↓ p- AKT	20, 40, 60 and 80 μM	[139]
	SMMC-7721	Induces apoptosis	↑ G0/G1-phase arrest, ↓ % age of cell s in G2/M-phase, ↑ %age of early apoptosis, late apoptosis, and total apoptosis, ↑ caspase 8, ↓ Bcl-2, ↑ intracellular autophagosomes, ↑ LC3B ↑ BECN1 mRNA, ↑ conversion of LC3B-I to LC3B-II, ↑ Beclin1	0, 12.5, 25, 50, 100, and 200 µM	[140]
	HepG2, HLF, and HAK-1B	Induces apoptosis	↓ cancer cell proliferation, ↑ cleaved caspase-8, caspase-3, caspase-7 and PARP, ↑ Fas/CD95 expression, ↓ p-STAT3s, ↓ Tyr705-phosphorylated STAT3, ↓ Ser727-phosphorylated STAT3, ↓ cyclin D1, ↓ survivin, ↓ Bcl-xL, ↓ VEGF, ↓ Tyr-phosphorylated CDK5	0, 10, 20, 50 μM	[141]
Liver	HepG2	Induces apoptosis	↑ frequency inhibiting HepG2 cell proliferation than free luteolin, ↑ enhance the uptake of drugs by cells, ↓ Bcl-2 and ↑ LDH	Luteolin-loaded PD-L1 targeted stealth PLGA/Liposomes (5.0 mg luteolin)	[142]
	MHCC97-H, HepG2,PLC/PRF/5, Hep3B, HEK293	Induces apoptosis	↑ inhibitory impact of VVIL-24 on liver cancer cells viability, ↑ IL-24 gene expression, ↑ apoptosis frequency, ↑ cleaved PARP, cleaved caspase-3, cleaved caspase-8, ↓ procaspase-3 and procaspase-8, ↓ XIAP	VV-IL-24 (4 MOI) and Luteolin (5 µg/mL)	[143]
	Huh7 andHep3B	Induces apoptosis and autophagy	↓ cell viability, ↑ apoptotic bodies, ↑ LC3-II, ↓ p62, ↑ DR5, ↑ cleaved caspase-3 and cleaved caspase-8	0, 5, 10 and 20 µM	[144]
Bladder	T24, 5637 with a p53 mutation and RT-4 with wild-type p53	Induces apoptosis	↑ G2/M arrest, ↑ p21Waf1/Cip1, ↑ p27Kip1, ↓ cyclin A and D1, ↓ phospho(p)-Akt, ↓ phospho(p)-p70S6K, ↓ phospho(p)-S6, ↑ TRX1, ↓ Intracellular ROS	0, 1, 10, 25, 50, 100 µM	[145]
Colorectal	HCT 116 and SW 620 (Oxaliplatin resistant)	Induces apoptosis	↑ Nrf2, ↑ NQO1, ↑ HO-1, ↑ GST α1/2, ↓ reduced glutathione, ↑ chemotherapeutic potential of cisplatin, oxaliplatin and doxorubicin	1, 5 and 10 μM	[146]
Choriocarcinoma	JAR and JEG-3	Induces apoptosis	↓ Proliferation and viability of cancer cells ↑ apoptosis frequency, ↑ loss of mitochondrial membrane potential, ↓ p-AKT, ↓ p-P70S6K, ↑p-GSK3β, ↓ AKT, ↑ERK1/2, ↓ PI3K/AKT and ERK1/2 signaling pathways ↓ SREBP1, ↓ SREBP2, ↓ SCAP mRNAs, ↓ p-mTOR, ↓ lipogenic genes	0, 5, 10 and 20 μM	[147]
Cervical	HeLa	Induces apoptosis	↓ methylation of crucial tumor suppressor genes like APC, BRCA1, CDH13, CDKN2, MGMT, MLH1, RARB, RASSF1 and TIMP3, ↓ global DNA methylation, ↓ DNMT activity, ↓ Histone deacetylation activity, modifies the expression of various chromatin-modifying enzymes, ↓histone methyl transferases such as ASH1L, WHSC1, SU2V40H1, ↓ HAT activity	5, 10, and 20 µM	[148]

**Table 3:** Anticancer effects of luteolin via modulation of metastatic proteins and miRNAs. Arrows designate up (↑), downregulation (↓) of the molecules.

Type of Cancer	Cell Line	Effects	Mechanism	Concentration	References
Melanoma	C918 and OCM-1	Suppress metastasis	↓ proliferation, adhesion, migration and invasion, ↓ MMP-2, ↓ MMP-9, ↓ PI3K/Akt signaling pathway, ↓ fluorescence intensity of F-actin, ↓ inhibit cellular F-actin aggregation, ↓ p-PI3K P85, ↓ p-Akt expression	0, 2.5, 5, 10, 20, 40 µM	[105]
	A375	Suppress metastasis	↓ MMP-2, ↓ MMP-9, ↑ TIMP-1 and TIMP-2, ↓ p-AKT1, ↓ p-PI3K, ↓ PI3K/AKT pathway	0, 10, 15 and 20 μM	[124]
	A375 and B16-F10	Suppress metastasis	↓ migratory, invasive, adhesive, and tube-forming potential, ↓ EMT, ↑ E-cadherin, ↓ N-cadherin and vimentin, ↓ p-Akt, ↓ HIF-1α, ↓ VEGF-A, ↓ p-VEGFR-2, ↓ MMP-2, ↓ MMP-9	5, 10, and 20 μM	[63]
Glioblastoma	U-87 MG and T98G	Inhibits migration	↓ Cdc42 (cell division cycle 42), ↓reduced PI3K/AKT activation, ↓ proteaosome pathway, ↑ Cdc42 proteolysis	15 and 30 μM	[123]
Breast	MDA-MB-231, MDA-MB-486, 4T1 and BT-549	Inhibits metastasis	↓ proliferation and metastasis, ↓ AKT/mTOR signaling pathway, reversed the epithelial-mesenchymal transition (EMT), ↓ MMP9, ↓ AKT/mTOR, ↑ H3K27Ac and H3K56A, ↑ p-AKT and p-mTOR proteins	0, 10, 20, and 30 μM	[149]
	MDA-MB-231, MCF10A, 4T1	Inhibits metastasis, and recurrence	↓ cell migration proliferation and colony formation, ↓ YAP/TAZ transcriptional activity and nuclear localization, ↓ EMT, ↓ fibronectin, N-cadherin, and vimentin, ↑ E-cadherin, ↓ CTGF and CYR61	0, 5, 10, 20, 40, 80 μM	[150]
	MDA-MB-231	Suppress metastasis	↓C-X-C, chemokine receptor type 4 (CXCR4), ↓ MMP-2, ↓ MMP-9	--	[122]
	MDA-MA-231 and BT5-49	Suppress metastasis	↓ cell invasion, ↓ β-catenin expression, reorganization of cytoskeletal protein F-actin in the cytoplasm, ↑ E-cadherin, ↑ claudin, ↓ N-cadherin ↓ vimentin, ↓ Snail, ↓ Slug,	0, 10, 30 and 100 μM	[61]
Lung	A549	Inhibition of cell migration	↓ cell motility and migration, suppression of MEK-ERK pathway by PD98059 significantly reversed luteolin-inhibited cell migration, ↑ E cadherin, ↓ N-cadherin	0–100 μM	[151]
Oesophageal	TE-1	Suppress metastasis	↓ stem-like properties of PTX-resistant cancer cells, ↓ SOX2, ↓ PI3K/AKT, ↓p-AKT(S473), ↓ UBR5 expression (ubiquitin E3 ligase that promotes SOX2 degradation), ↓ PTX-resistant cancer cell migration and invasion by blocking epithelial-mesenchymal transition (EMT)	--	[152]
Hepatocellular	HepG2, Huh7	miRNA regulation	↑ miR-6809-5p (miR-6809-5p targets flotillin 1 (FLOT1) in HCC), ↑ FLOT1 prevented miR-6809-5p-mediated growth suppression. Multiple signaling pathways including Erk1/2, p38, JNK, and NF-κB/p65 were inactivated by miR-6809-5p overexpression or FLOT1 downregulation	10 μM	[153]
Gastric	MKN45 and BGC823	Inhibits metastasis	↓ cell migration and invasion, ↓ lung metastasis, ↓ Cyclin D1, ↓ Cyclin E, ↓ Bcl2, ↓ MMP2, ↓ MMP9, ↓ N-cadherin, ↓ Vimentin ↓ Notch1, ↓p-PI3K, ↓ p-AKT, ↓ p-mTOR, ↓ p-ERK, ↓p-STAT3 ↑ p-P38 signaling, ↑ p21, ↑ Bax, ↑ E-cadherin,	20 and 40 μM	[82]
	MKN45 and BGC823	miRNA regulation	↑ miR-139, ↑ miR-34a, ↑ miR-422a, ↑ miR-107 (tumour suppressor), ↓ miR-21, ↓ miR-155, ↓ miR-224, ↓ miR-340 (oncogenes)	20 and 40 μM	[82]
Colorectal	HT-29, SW480, SW620 and LoVo	Suppress metastasis	↓cells migration and invasion, ↓ MMP-2, ↓ MMP-3, ↓MMP-9, ↓ MMP-16	0, 10, 50 and 100 µM	[92]
	HT-29, SW480, SW620 and LoVo	miRNA regulation	↑ miR-384 and ↓ PTN expressions, miR-384 inhibitor partially reversed the inhibition of cells migration and invasion induced by luteolin	0, 10, 50 and 100 µM	[92]

**Table 4:** Anticancer effects of luteolin based on in vivo studies. Arrows designate up (↑), downregulation (↓) of the molecules.

Type of Cancer	Animal Models	Effects	Mechanisms	Dosage	Duration	References
Osteosarcoma	BALB/c nude mice xenografted with MG 63 5 × 10^6^ cells	Inhibited tumor growth	↓ tumor size and growth, ↑ anti-tumor effect in combination with doxorubicin, ↑ miR-384, ↓ PTN, β-catenin and P-glycoprotein	2 mg/kg doxorubicin + 30 mg/kg luteolin	28 days	[126]
Breast	BALB/c nude mice xenografted with 4T1 cells	Inhibited the tumor growth	↓ final tumor volume and weight, ↓ YAP/TAZ expression	40 mg/kg	18 days	[150]
Lung	Patient-derived xenograft mouse model with SCID mice	Inhibited tumor growth	↓ tumor growth and weight, ↓ Ki-67, ↓ p-Limk1/2 and p-cofilin expression	100 mg/kg	59 days	[38]
	Nude mice model with H358 xenografts	Inhibited tumor growth	↓ tumor volume and size,↓ tumor weight, ↓ lunglesions, ↑ %age CD8+ T cells in blood, spleen, or tumor was increased, ↑ IFN-γ, TNFα,and Granzyme B	30 mg/kg of apigenin or luteolin	21 days	[132]
	Lewis lung carcinoma model with C57BL/6J mice	Inhibited tumor growth	↓ tumor volume and size, ↓ tumor weight, ↓ lung lesions, ↑ %age CD8+ T cells in blood, spleen, or tumor was increased, ↑ IFN-γ, TNFα,and Granzyme B	30 mg/kg of apigenin or luteolin + anti-PD-L1 mAb (10 mg/kg)	21 days	[132]
	KRASLA2 mice model	Inhibited tumor growth	↓ tumor volume and size, ↓ tumor weight, ↓ lunglesions, ↑ %age CD8+ T cells in blood, spleen, or tumor was increased, ↑ IFN-γ, TNFα, and Granzyme B	30 mg/kg of apigenin or luteolin	21 days	[132]
	Nude mice model with H460 xenografts	Suppressed tumor growth	↓ tumor volumes and tumor weights, ↑ inflammatory cell infiltration, ↑ clear cell death characteristics and phenotype, ↑ TUNEL-positive cells were, ↓ Ki67-labeling index, ↑ miR-34a-5p, ↑P53 and P21, ↓ MDM4	50, 100, and 200 mg/kg/day)	15 days	[81]
	C57BL/6 Nrf2+/+ and Nrf2/ mice xenografted with A549 tumor cells (1 × 10^7^ cells)	Inhibited tumor growth	↓ NQO-1 expression, ↓ protein level of NQO1 AKR1C, HO-1, and GSTm1	cisplatin only (5 mg/kg), luteolin only (40 mg/kg), or a combination of cisplatin (5 mg/kg) and luteolin (40 mg/kg).	35 days	[154]
Gastric	BALB/c male nude mice xenografted with MKN28 cells	Inhibited the tumor growth	↓ tumor volume and tumor eight, ↓ β-catenin, ↓ Notch1, ↓ Ki-67 expression, ↑ TUNEL staining	--	4 weeks	[134]
	Human tumor xenograft (PDTX) models of gastric cancer (BALB/c nude mice)	Inhibited the tumor growth	↓ cMet protein, ↓ MMP9, ↓ p-cMet	10 mg/kg	30 days	[135]
Hepatocellular	BALB/c nude mice xenografted with 4 × 10^6^ MHCC97-H cells	Inhibited tumor growth	↓ tumor growth, ↑ IL-24 protein, ↓ CD31, ↓ Ki67 staining, ↑ protein level cleaved caspase-3, ↑ cytopathic effect	luteolin (50 mg/kg) alone, intraperitoneal injection; VV-IL-24 (2 × 10^7^ plaque-forming units) and their combination	35 days	[143]
	BALB/c athymic nude mice injected with HAK-1B cells	Inhibited the tumor growth	↓ tumor volume, ↓ Tyr705-phosphorylated STAT3, ↓ Ser727-phosphorylated STAT3, ↓ cyclin D1, ↓ VEGF, ↑ Fas/CD95, ↑ cleavage in caspase-7	50 or 200 ppm	6 weeks	[141]
Pancreatic	Female Syrian golden hamsters injected with subcutaneousinjections of BOP	Inhibited pancreatic carcinogenesis	↓total cholesterol, ↑ amylase, ↓ incidence and multiplicity of PDACs, ↓ progression of neoplastic lesions, ↓ Ki-67 labeling index ↓ lesions, ↓ DPYD ↓ pSTAT3 signaling	100 ppm	6 weeks	[136]
	SCID mice xenografted with 1.65 × 10^6^ SW1990 cells	Inhibited tumor growth	No pathological changes in these normal tissues compared with the vehicle-treated group, did not produce remarkable weight loss of mice	75 mg/kg and 150 mg/kg	2 weeks	[137]
Bladder	KSN nude mice xenografted with 5 × 10^4^ BC31 cells	Inhibited the tumor growth	↓ toxic effect, tumor volumes, ↓ Ki67-labeling index, ↑ TUNEL-positive cells, ↓ proliferation of cancer, ↑ apoptosis frequency, ↑ p21-positive cells	100 ppm	5 weeks	[145]
Colorectal	C57BL/6 Nrf2+/+ and Nrf2−/− mice	Inhibited the tumor growth	↑ Nrf2 and NQO1, HO-1, GST α1/2, ↓ reduced glutathione	40 mg/kg	14 days	[146]

## Data Availability

Data sharing not applicable to this article as no datasets were generated or analyzed during the current study.
